# Electroforming of Personalized Multi-Level and Free-Form Metal Parts Utilizing Fused Deposition Modeling-Manufactured Molds

**DOI:** 10.3390/mi15060734

**Published:** 2024-05-31

**Authors:** Hazem Hamed, Sayedmohammadali Aghili, Rolf Wüthrich, Jana D. Abou-Ziki

**Affiliations:** 1 Department of Mechanical and Manufacturing Engineering, University of Ontario Institute of Technology (Ontario Tech University), 2000 Simcoe St. N., Oshawa, ON L1L 0M7, Canada; hazem.hamed@ontariotechu.net; 2 Department of Mechanical Industrial and Aerospace Engineering, Concordia University, 1455 De Maisonneuve Blvd. W., Montreal, QC H3G 1M8, Canada; mohamad.aghili@concordia.ca

**Keywords:** personalization, electroforming, metal additive manufacturing, fused deposition modeling (FDM), mold

## Abstract

Adapting to the growing demand for personalized, small-batch manufacturing, this study explores the development of additively manufactured molds for electroforming personalized metal parts. The approach integrates novel multi-level mold design and fabrication techniques, along with the experimental procedures for the electroforming process. This work outlines design considerations and guidelines for effective electroforming in additively manufactured molds, successfully demonstrating the production of composite metal components with multi-level and free-form geometries. By emphasizing cost efficiency and part quality, particularly for limited-thickness metal components, the developed technique offers distinct advantages over existing metal additive manufacturing methods. This approach establishes itself as a flexible and durable method for metal additive manufacturing, expanding the scope of electroforming beyond traditional constraints such as thin-walled hollow structures, 2D components, and nanoscale applications.

## 1. Introduction

In contemporary manufacturing, a notable shift is observed towards the fabrication of smaller batches of personalized products [1]. This transformation resonates with the principles of just-in-time manufacturing and addresses the escalating demand for customization, rapid prototyping, and the growing demand for the personalization concept. Diverging from mass customization, personalization entails an active engagement of customers in product design, often resulting in limited batch sizes [2]. However, this trend poses imposing challenges to conventional mass production methodologies, ill-equipped to navigate the intricacies of niche, personalized production. The production of personalized items involves additional expenses compared to mass-produced and customized products, mainly due to the active engagement of customers in the design process. This renders cost-effective small-batch production a significant challenge [3].

To tackle this challenge, digital manufacturing, often known as Industry 4.0, emerges as a promising solution [4]. In digital manufacturing, cyber-physical systems offer the needed flexibility. These systems enable swift and efficient adjustments of production lines to accommodate customer design inputs. They integrate various functions, including computer-aided design (CAD), simulation, visualization, and analytics [5]. The importance of digital manufacturing becomes more apparent as product complexity and manufacturing processes grow more intricate. Within this context, Additive Manufacturing (AM) processes emerge as a promising technology for personalized small-batch-size production [6]. These processes play a significant role by essentially providing “complexity for free” through virtual means.

Additive Manufacturing (AM) stands out for its proficiency in intricate part design and rapid lead times [7]. Moreover, it upholds cost-efficiency for single, complex, or customized items, maintaining a consistent cost per unit regardless of volume or complexity. However, despite these advantages, current metal AM techniques face several challenges. Material choice, especially in the metal category, remains somewhat limited. Present technology only allows the printing of a select range of materials, thus hindering the full utilization of its capabilities. Additionally, post-processing steps are often imperative to achieve mechanical attributes and surface quality comparable to those attained with traditional manufacturing methods [8]. This supplementary phase extends production time and introduces complexity to the process.

Electroforming, traditionally employed for shaping parts on mandrels, has recently gained attention for the additive manufacturing of metal components [9,10,11]. It involves the process of generating or duplicating material through electrodeposition onto a mandrel or mold, which is subsequently detached from the deposited material. Electroforming offers notable advantages over traditional manufacturing techniques, facilitating the production of parts that may be difficult or even impossible to fabricate using conventional methods. These advantages encompass high dimensional accuracy and consistency, low surface roughness, atomic-scale deposition, adjustable mechanical properties, and cost-efficiency [12,13,14].

A considerable portion of electroforming research centers on the LIGA process, with numerous publications investigating applications at the nanoscale and optimizing process parameters to enhance the quality of electroformed components [15]. However, a notable gap exists in research regarding the utilization of electroforming at the microscale, particularly for depositing thick layers. The challenges associated with electroforming thick deposits are compounded by the intricate nature of residual stress, which can compromise structural integrity. Additionally, the restricted ion transportation within micro-channels poses further obstacles to achieving successful thick deposits [16].

A novel concept has emerged proposing the utilization of additive manufacturing as a cost-effective and time-efficient approach for producing personalized mandrels and molds, followed by electroforming or electroplating [17,18]. This indirect manufacturing process presents a promising solution for precision metal parts’ fabrication, boasting advantages such as low energy consumption and minimal equipment investment costs, particularly for specific designs. Additionally, this fabrication technique harnesses the potential to create intricate mold geometries through additive manufacturing while simultaneously manufacturing multiple parts through electroforming, all without incurring additional costs associated with design alterations prompted by customer preferences.

Moreover, electroforming, as a subsequent step, offers superior surface quality compared to direct metal printing, potentially mitigating concerns regarding extensive post-processing [19,20]. Angel et al. [21] showcased the production of a solenoid inductor with low-frequency inductance through Fused Filament Fabrication (FFF) and electrodeposition. Similarly, Aghili et al. [22] were able to fabricate a precise 2D flexure mechanism utilizing electroforming of additively manufactured molds. Zheng et al. [18] successfully demonstrated the fabrication of hollow copper-net shape parts using FDM-printed mandrels and electroforming, achieving a high uniformity and dimensional accuracy in thin-walled multi-thickness structures.

In a separate study, Matsuzaki et al. [23] examined the viability of producing multi-material structures consisting of resin and copper using a combination of FFF and electroforming techniques. Phull et al. [24] delved into the creation of metal tooling by integrating FDM with nickel electroforming. Additionally, McCarthy and Williams [25] combined Selective Laser Sintering (SLS) with electroforming to manufacture complex thin-walled metal structures, producing cellular nickel structures that were challenging to achieve with other manufacturing methods. The primary process involved printing the desired mandrel or mold, activating the designated area for forming, conducting the electroforming process, and ultimately separating the final part from the mandrel or mold.

However, the current utilization of this technique is primarily restricted to the production of thin-walled hollow structures, extruded (2D) metal structures, and multi-material parts combining plastic and metal. The present study aims to establish a series of design protocols for developing modular molds, facilitating the electroforming of multi-level and free-form metal components, specifically with thicknesses exceeding 1000 µm. This process involves employing Fused Deposition Modeling (FDM) to fabricate the molds, followed by electroforming to additively manufacture the desired metal components. The research is structured to explore the potential for achieving mass personalization of metal structures across both macro- and microscales using this cost-effective additive manufacturing approach.

## 2. Methodology

The study focuses on utilizing additive manufacturing to produce molds, facilitating the electroforming of diverse geometries, including multi-level objects. Within the realm of multi-level configurations, parts display three axes with limited variation in the third axis, featuring multiple flat features at varying depths. In contrast, free-form parts exhibit intricate shapes and features across all three dimensions.

The primary objective was to introduce a modular mold design that adhered to specific procedures suitable for electroforming. Prioritizing economic viability, the design eliminated the need for expensive masks and ensured conductivity through an affordable solution. Subsequently, an experimental process was developed, encompassing mold design and fabrication. This mold was then utilized in the electroforming of the desired part. The overall fabrication process emphasized cost-effectiveness, repeatability, reliability, and automation potential.

### 2.1. Mold Preparation and Electroforming Procedures

**Figure 1:**
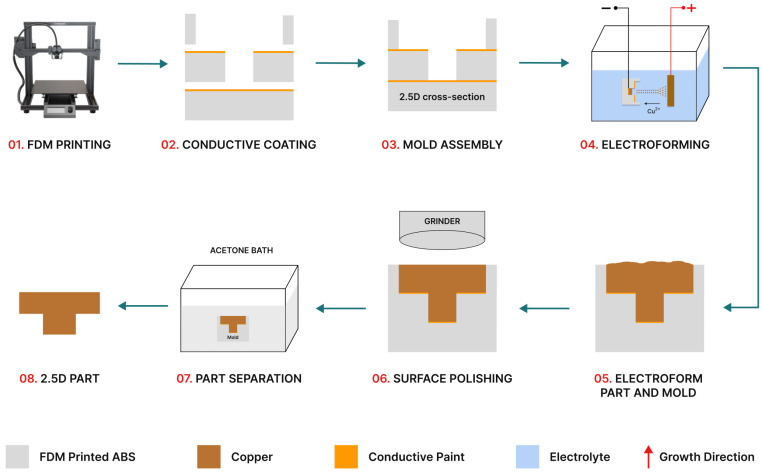
A diagram illustrating the procedural steps involved in producing metal parts through electroforming.

To establish a solid foundation for electroforming within an additive manufacturing mold, several critical steps must be undertaken. These include applying a conductive coating, ensuring a solid bonding of mold components during assembly, and implementing a non-destructive technique for part separation. One of the main objectives was to devise an experimental process that is cost-effective, reliable, repeatable, and has automation potential, covering all stages from mold design and fabrication to the electroforming of the desired part. Figure 1 offers a visual representation of the procedural steps involved in this endeavor.

#### 2.1.1. Fused Deposition Modeling (FDM)

The mold components were 3D-printed using an FDM printer, with Acrylonitrile Butadiene Styrene (ABS) material serving as the printing filament. ABS was chosen for its smooth surface finish and compatibility with acetone bonding. The quality and precision of the FDM part are critical for a successful electroforming process, capable of sub-micron replication [26]. Any flaws in the initial FDM part affect the final electroformed product. To ensure the best possible surface roughness and geometry, the FDM printing parameters were specifically optimized for ABS. The printing parameters employed in this study are illustrated in Table 1.

To ensure proper adhesion and minimize warping of the printed layers, several measures were during the printing process. The build plate was maintained at 95 °C, the fan was disabled (set to 0% speed), and a printing glue was applied to enhance adhesion. Additionally, a 3 mm brim was added to all printed parts. A critical parameter in the FDM process is the printing layer thickness, which was set to 0.1 mm. This choice was informed by the understanding that layer thickness significantly influences approximately 85% of the overall accuracy of FDM-printed parts compared to other parameters. It has been established that for FDM parts using ABS, optimal dimensional accuracy is achieved when the layer thickness ranges between 0.1 and 0.2 mm [27].

The printing speed was maintained at 30 mm/s, and the nozzle extruder temperature was set to 245 °C, in accordance with manufacturer recommendations. A 50% infill density was selected to mitigate geometrical deviations in the ABS printed parts [28]. Once printing was completed, the printer was allowed to cool down to ambient temperature, after which the parts were detached from the printing plate and the attached brim was removed. These steps were integral to the overall experimental process, ensuring precision and consistency in part production.

#### 2.1.2. Selection and Application of Conductive Coating

A silver-coated copper conductive paint was employed. The chosen conductive paint, identified as MG Chemicals 843AR Liquid, offered a high conductivity (3 × $10^{-4}$Ω·cm), rapid drying time, and strong adhesion to most plastics. Various painting techniques were experimented with and assessed to ascertain the most efficient method for creating a conductive printed layer. Both brush-painting and spray-painting methods were evaluated for comparison.

Brush painting presents the benefit of applying thicker paint coatings, which could potentially improve conductivity. However, spray painting offers several advantages, such as its rapid and automatable process, faster drying times, and minimal influence on the mold’s surface roughness. Moreover, it guarantees a more uniform and consistent paint application across all mold components.

A high-volume low-pressure (HVLP) spray gun with a nozzle tip diameter of 1.4 mm was utilized for applying the conductive layer, following the recommendations of the paint manufacturer. The paint was thoroughly mixed before dispensing it through the HVLP spray gun using an air compressor line, with the air pressure regulated within the range of 30–35 PSI. After each coat, a re-coating time of 3 min was allowed for the paint to cure before applying another layer. To achieve a surface characterized by a high electrical conductivity, a minimum of 3 coats of the conductive paint was recommended. The electrical resistance of the mold components was evaluated using a multimeter, resulting in resistance readings ranging from 0.3 to 0.6 ohms (Ω).

#### 2.1.3. Bonding of Mold Layers

Several techniques exist for bonding the layers including glue bonding and hot-pressing. The chosen method for bonding the mold components, however, involved chemical bonding using acetone, selected for its simplicity and ease of application. This approach eliminates the need for heating or applying mechanical pressure during mold assembly. The combination of ABS as the selected mold material and acetone as a polar solvent effectively addressed several challenges in this process. When acetone comes into contact with ABS material, it initiates the formation of a sticky outer surface. This property was leveraged to bond the printed ABS components together seamlessly.

Acetone was applied to the rear surface of each layer and pressed against the corresponding layer for a few seconds to ensure robust bonding. However, it is crucial to use acetone cautiously for bonding the mold components, as excessive application can lead to the dissolution of the layer, potentially affecting its thickness. Following the bonding of the mold components, an additional step involved applying varnish to the outer surfaces of the mold to insulate them. This insulation prevented unintended deposition in areas outside the mold’s intended surfaces during the electroforming process.

#### 2.1.4. Electroforming Setup and Preparation

Once the mold assembly was completed, it underwent preparation for the electroforming process. The electroforming process took place in a solution comprising sulfuric acid and copper sulfate, without the incorporation of any additives. Furthermore, a magnetic rod was employed to steer the electrolyte, ensuring its uniform distribution and enhancing the surface finish [29].

**Figure 2:**
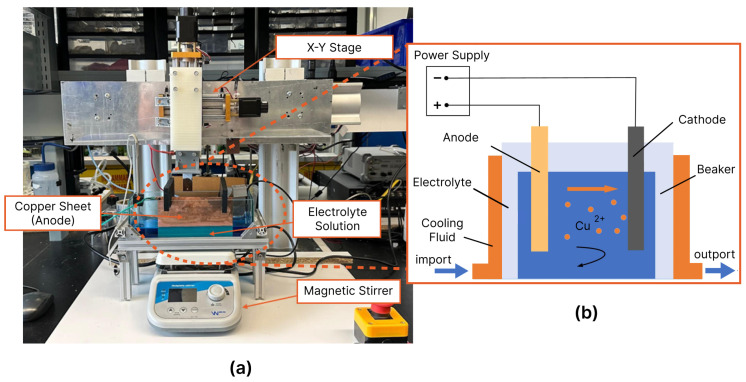
(**a**) The experimental setup utilized for electroforming in this study. (**b**) A schematic illustrating the basic principles underlying the electroforming process.

During the electroforming process, the mold assembly was subjected to reciprocating movement by an X-Y stage, maintaining parallel alignment with the counter electrode. A pulsed deposition technique was employed, alternating between deposition pulses (at −150 mV vs. Cu) and polishing pulses (at +100 mV vs. Cu). To effectively manage the layer thickness in each pulse, a consistent deposition charge was maintained, as there exists a direct correlation between deposit thickness and the applied charge. The electroforming setup is depicted in Figure 2.

Instead of employing a fixed-time deposition method, a fixed-charge deposition approach was utilized. Deposition pulses persisted until a charge of 1.33 C/cm² was reached in the electrochemical circuit, equivalent to a thickness of approximately 1 µm. Conversely, polishing pulses endured for 10% of the deposition charge duration. The deposition thickness is predominantly influenced by two key parameters: the number of pulses and the surface area where the deposit is intended to grow. This pulsed deposition process is instrumental in achieving high-quality thick deposits and averting the formation of voids in the deposited material, as evidenced by previous research [30].

#### 2.1.5. Surface Polishing

Sandpapers with a grit size of 220 were utilized to gradually remove material until the desired intersection was revealed. Following this, the sample underwent multiple rounds of polishing using 600 grit sandpapers to attain a smoother surface finish. This iterative process refines the surface, improving its smoothness and eliminating any lingering imperfections.

#### 2.1.6. Part Separation

Upon completing the polishing phase, immersion of both the mold and the electroformed part in acetone ensued, enabling the effortless separation of the electroformed part from the mold. Notably, acetone exhibited remarkable efficacy in fully dissolving ABS within a few hours, playing a pivotal role in parting the mold. As a result, the electroformed part could be safely extracted from the mold without incurring any damage.

### 2.2. Mold Preparation and Fabrication

To effectively serve its purpose, the mold needed to be designed to support the growth mechanism inherent in the electroforming process while being versatile enough to produce a diverse range of intricate geometries, particularly those associated with multi-level and free-form structures. Additionally, automation potential was factored into the mold design and manufacturing processes to enable the production of mass-personalized parts in various configurations. Thus, the mold design was standardized for fabricating different parts with various shapes and features.

A negative mold configuration was chosen to correspond with the desired shape of the end product. This mold design adopted an open-surface configuration, facilitating smooth metal ion transfer throughout the electroforming process. Moreover, Fused Deposition Modeling (FDM) was employed for the mold fabrication because of its simplicity, cost-effectiveness, and ability to handle intricate geometries. Thereby, enabling the production of modular molds with varying levels of complexity.

**Figure 3:**
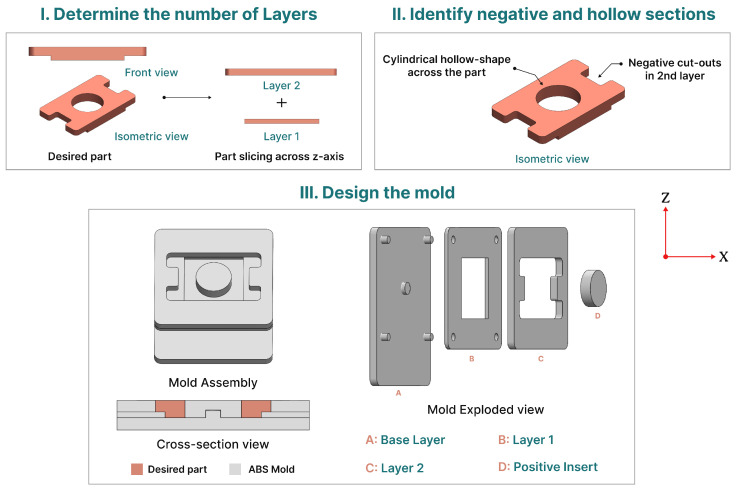
A diagram depicting the methodology and design considerations for electroforming molds.

The first step in mold design involved defining the geometry of the desired part. This process included assessing the part’s dimensions along the z-axis to determine the necessary number of mold layers. By focusing on changes in width along this axis, any significant variations prompted the creation of additional layers. For example, in the part depicted in Figure 3, a single change in width along the z-axis led to the division into two layers.

In the subsequent step, the process entailed identifying negative cutouts and hollow sections within each layer of the mold. Given the mold’s negative configuration, it became imperative to introduce corresponding positive counterparts to accommodate these cutouts and hollow sections in the desired part. For example, if the illustrated part featured two negative cutouts in its second layer and a cylindrical hollow shape spanning its entire depth, the mold design incorporated a positive shape with matching dimensions into the second layer. Furthermore, to complement the specified hollow shape, a cylindrical positive insert was introduced, extending throughout the height of the desired part.

Once the number of layers and the negative cutouts in the desired part were determined, the mold design process commenced. Every mold necessitates a foundational base layer, uniformly coated with a conductive paint, typically copper or silver. To ensure consistency in the spraying process, a flat rectangular part was chosen as the base. The subsequent layers added atop the base layer defined the mold’s shape and, consequently, the form of the part. For the desired part, three layers were required: a base layer, layer one, and layer two. It was essential that layer one aligned with the dimensions of its corresponding section in the desired part.

The copper electroforming process commenced on the base layer, gradually building up until it reached the required thickness of layer one on the mold. Subsequently, copper growth continued at the base of layer one until it achieved the necessary thickness of the second layer. The presence of the positive insert prevented copper deposition on the cylindrical cutout, maintaining its intended shape.

Finally, the base layer was equipped with four locator pins and a central positioning pin. These locator pins ensured the precise alignment and positioning of all mold layers during assembly. Conversely, the central pin was specifically intended for positioning the positive insert that interfaced with the hollow cylindrical shape. A schematic illustrating and summarizing the mold design development is provided in Figure 3.

## 3. Fabricating Metal Components via Electroforming

Electroforming has traditionally been associated with the production of thin-walled hollow structures, two-dimensional (2D) part manufacturing, and applications at the nanoscale. Additionally, its utilization has typically been confined to the manufacturing of low-profile parts, typically below 1000 μm in thickness. However, this section seeks to explore the fabrication technique that might extend the capabilities of electroforming beyond these limitations. Specifically, it investigates the creation of both multi-level objects and free-form metal components through the electroforming process, utilizing additively manufactured molds.

Electroforming using FDM molds offers a promising solution for mass and batch personalization of metal parts. FDM is widely favored in various industries due to its numerous advantages. These include low maintenance costs, a broad range of available materials, cost-effectiveness for creating complex geometries, and ease of operation with minimal supervision [31]. In this process, the customer provides the desired part design, and the mold is subsequently designed based on the specified geometry. The required number of molds can then be rapidly produced using FDM printing. These molds are then subjected to electroforming to fabricate parts that meet the necessary specifications. This method facilitates the fabrication of personalized metal components using a cost-effective approach compared to the available direct metal printing techniques. By leveraging FDM molds for electroforming, it is possible to achieve intricate geometries and high-quality surface finishes at a lower cost and with greater flexibility in production, which is ideal for personalized and small-batch manufacturing.

**Figure 4:**
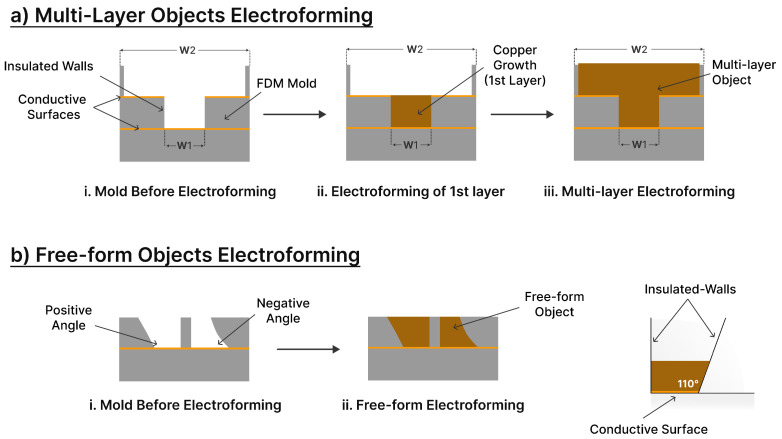
A schematic showing the electroforming growth mechanism of the (**a**) multi-level objects and (**b**) free-form objects.

The electroforming process for multi-leveled objects relies upon the mold’s geometry. Therefore, a technique was introduced to incorporate multiple conductive surfaces. Initially, deposition occurred solely on the primary step of the structure. Once the deposit reached the same level as the conductive surface on the secondary step, an electrical connection formed between the top surface of the copper deposit and the secondary conductive surfaces. This method guaranteed complete coverage of the entire surface by an electroformed layer, resulting in enhanced surface uniformity and a consistent deposition layer that replicated the mold’s contours. In the case of free-form objects, electroforming relied on altering the mold surface angles and introducing curves to facilitate the electroforming of the desired structure. Figure 4 illustrates the growth mechanism of electroforming for both multi-level structures and free-form objects.

During the experimentation of electroforming in AM molds, it was observed that structure growth occurred only within specific wall angles. Various angles were tested, revealing an issue of disconnection when the angle was excessively steep or shallow. This disconnection arose from the increased non-conductive distance, causing the deposit to fail in completing the intended structure. Angles ranging from 20° to 110° proved optimal when electroforming free-form structures in the AM molds. Consequently, free-form structures may feature both negative and positive angles, as well as curvatures as depicted in Figure 4.

Furthermore, the methodology used to separate the mold components simplified the electroforming of intricate shapes. Employing a multi-layered mold streamlined the application of conductive paint and the assembly of mold components, eliminating the complexities of selectively applying paint to specific areas while avoiding others. This approach also removed the need for expensive masks to apply paint in particular mold regions. As a result, the technique facilitated the electroforming of multi-level and free-form components without substantially complicating mold manufacturing. Moreover, standardizing the mold design and fabrication process enabled the parallel production of multiple parts.

To assess the effectiveness of this approach in producing intricate structures, two specific applications were chosen for evaluation. Firstly, a center wheel was manufactured to exemplify a multi-leveled object configuration. This wheel exhibited intricate details, such as micron-sized teeth, providing a comprehensive demonstration of the method’s capabilities. Additionally, a toroidal propeller was created to showcase how a manipulation of angles enabled the production of free-form structures using the electroforming process.

### 3.1. Multi-Level Objects

The first application chosen to demonstrate this fabrication method was a center wheel, selected for several reasons. Primarily, a center wheel possesses a complex geometry with micron-sized teeth arranged around its diameter, alongside hollow curved sections at its center. Additionally, this component showcases varying thicknesses throughout its depth, contributing to its classification as a multi-level object. Therefore, it served as an ideal test case for evaluating the effectiveness of the proposed fabrication approach in producing intricate multi-leveled structures.

Moreover, traditional methods for manufacturing high-precision center wheels in the industry involve a series of complex and costly processes, as discussed by Watkins R. [32]. These conventional techniques are not only time-intensive but also require significant labor. In contrast, the application of electroforming in molds produced by FDM printing offers a promising alternative. This approach presents a cost-effective solution that minimizes resource expenditure and reduces the time required for production compared to traditional methods.

**Figure 5:**
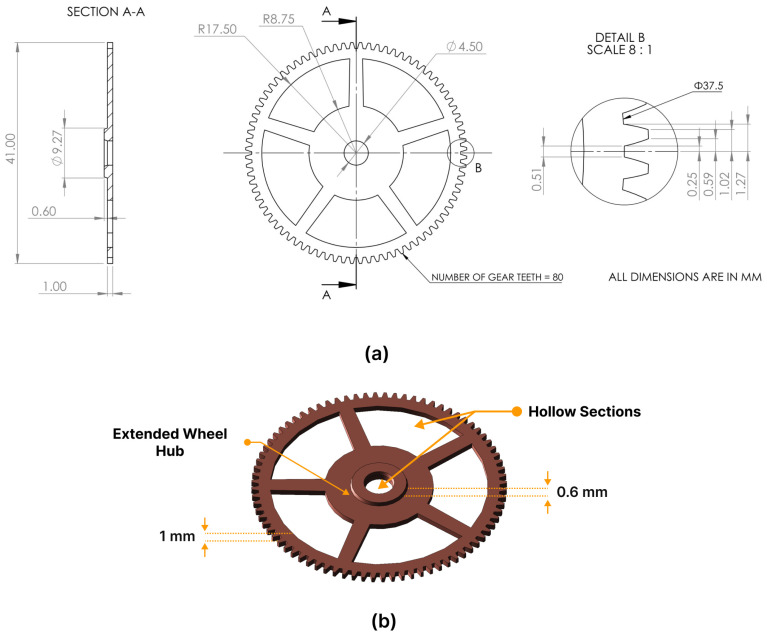
(**a**) An engineering drawing presenting the specifications and dimensions of the center wheel. (**b**) A 3D CAD model of the center wheel.

The center wheel, illustrated in Figure 5, was characterized by a configuration consisting of 80 teeth and a total diameter of 41 mm, with a root diameter of 37.5 mm. It had an overall thickness of 1.6 mm, composed of a 1 mm gear body and a 0.6 mm extended hub positioned at the center wheel’s midpoint, which had a diameter of 9.27 mm. Five spokes extended from a diameter of 17.5 mm outward to 35 mm. Additionally, the central hole of the gear had a diameter of 4.5 mm.

To develop an appropriate mold design for electroforming the center wheel, the methodology described in Section 2.1 was implemented. Employing only a substrate layer and a mask layer proved insufficient for producing the center wheel due to its multi-leveled structure, which entailed multiple thicknesses at varying depths. Therefore, integrating multiple layers above the substrate emerged as a viable solution for several reasons. This approach allowed for the creation of distinct flat features at different depths and addressed the requirement for certain layers to demonstrate conductivity during the coating of the ABS printed mold components, while others had to remain electrically isolated.

The mold was conceived with a three-layer design, incorporating positive inserts. This setup comprised a base layer, serving as the substrate. Positioned above was an intermediate layer, matching the 0.6 mm thickness of the center wheel’s extended hub. The intermediate layer also featured five positioning recesses designed to accommodate the positive inserts. The development of the extended hub began at the substrate level, gradually ascending to its intended thickness at the upper part of the intermediate layer.

Electroforming of the wheel body commenced from the intermediate layer, advancing towards the top layer. The top layer encapsulated the negative counterparts of the gear teeth. Furthermore, upon assembly atop the intermediate layer, the five positive inserts served as negative counterparts, delineating the contours of the hollow curved segments and forming the spokes.

The base substrate dimensions were 50 mm × 65 mm with a thickness of 2 mm. Within this framework, a 50 mm × 50 mm square was allocated as the core mold assembly zone. Additionally, an extended segment measuring 50 mm × 15 mm was integrated to guarantee secure mounting throughout the electroforming process. The substrate was equipped with four locator pins along its sides and one positioning pin in the center. These locator pins ensured an accurate alignment and positioning of the intermediate and mask layers during assembly.

**Figure 6:**
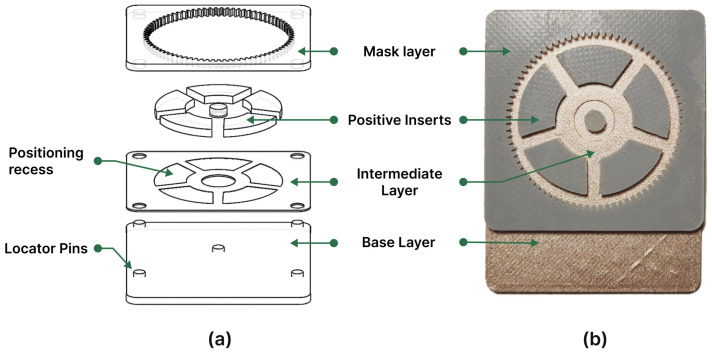
(**a**) A schematic representation of the components of the multi-level center wheel mold. (**b**) A photograph showing the fully assembled mold.

The central pin, however, served the purpose of positioning the center core, which functioned as a negative encounter for the hole in the middle of the center wheel. Figure 6 presents a schematic illustrating the center wheel mold components and the assembled mold after painting and assembly. The intermediate layer measured 50 mm × 50 mm and included four through holes at its corners, aligning with the pins in the base layer for straightforward positioning. Similarly, the mask layer incorporated four holes, serving as docking points for the pins, completing the assembly.

### 3.2. Free-Form Objects

The second application chosen for this study focused on the fabrication of a toroidal propeller, aiming to assess the process capability for additively manufacturing metal components with intricate features. This selection aimed to explore the feasibility of utilizing the presented electroforming process for creating complex three-dimensional geometries. The toroidal propeller exhibited a sophisticated free-form design, characterized by three closed loops and a central hub. Historically, the intricate shape of toroidal propellers has presented significant manufacturing challenges, leading to high production costs. This endeavor demonstrates the potential of altering mold surface angles and introducing curves to enable the fabrication of intricate free-form structures through electroforming.

The initial design of the toroidal propeller showcases intricate curvature patterns along its closed loops, where each loop functions as a blade for the propeller. Different designs have been put forth for the toroidal blade, spanning from two-blade arrangements to those comprising up to four blades. Despite variations in design, they all incorporate curved enclosed blades as a shared characteristic. This particular closed-form configuration aims to mitigate the detrimental impacts of swirling air currents generated at the blade tips while bolstering the propeller’s overall rigidity.

To overcome challenges associated with electroforming intricate blades featuring steep angles, adjustments to the original toroidal propeller design were necessary. These modifications aimed to facilitate a smooth growth within the mold during electroforming, thereby minimizing surface irregularities, disconnections in the deposit, and deviations from the intended structural shape. Particularly, alterations were made to the curved blades, reducing their steep angles while preserving the propeller’s functionality.

**Figure 7:**
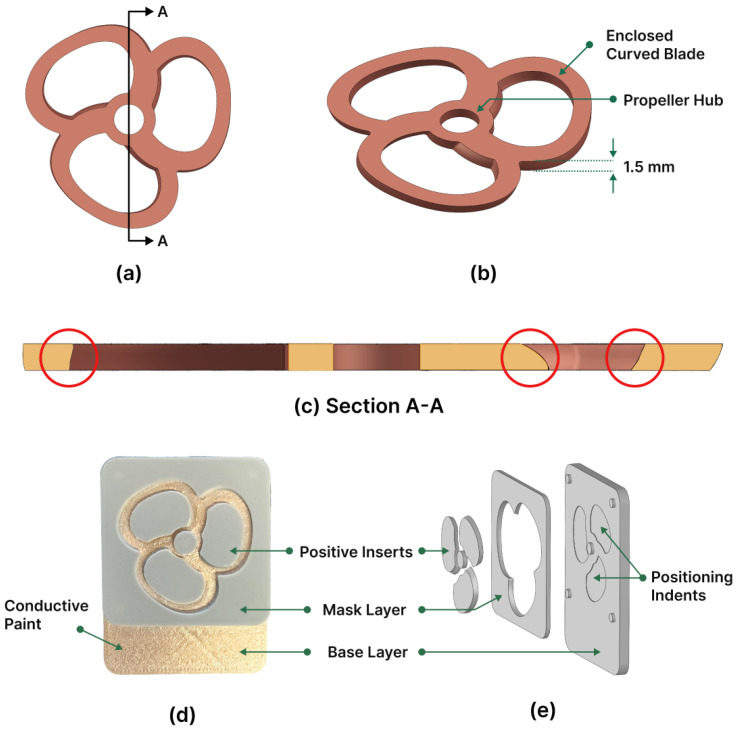
(**a**,**b**) CAD models illustrating the proposed design of the toroidal propeller. (**c**) A cross section depicting the intricate free-form shape of the toroidal propeller blades. (**d**) A photograph displaying the assembled mold, and (**e**) a CAD model showing the exploded view of the mold components.

The CAD model depicting the proposed toroidal blade design is presented in Figure 7b. This configuration comprised three closed loops, with each loop serving as an independent blade for the propeller. Furthermore, the propeller incorporated a hub designed for assembly onto a shaft. The intended thickness for the toroidal blade is 1.5 mm. These enclosed free-form blades featured diverse angles along their dimensions, ranging from 80° to 120°, and varied in width at different cross sections across the blade.

Figure 7d,e illustrate the mold CAD model and its components. Due to the consistent thickness of the toroidal propeller design across its cross section, only a single mask layer was required, alongside the base layer. To accommodate the cut-outs in the closed-loop blades, three positive inserts were devised, along with a cylindrical positive insert to correspond with the propeller hub hole. The intricate, multi-angle sections of the blades were seamlessly integrated into the positive inserts and the mask layer, leveraging the FDM capability to manage such intricate profiles. Additionally, the base layer incorporated three 0.1 mm deep positioning indents to ease the assembly of the positive inserts.

The mold fabrication process adhered to the previously delineated experimental technique in Section 2.2, with an additional step. Specifically, vapor smoothing of the mold components was introduced before the application of conductive paint and subsequent assembly. Vapor smoothing, also known as Chemical Vapor Smoothing (CVS), involves exposing printed parts to a vaporized solvent. The recent literature has documented several studies utilizing chemical solvents like acetone to enhance the surface quality of FDM-printed ABS parts [33,34]. This process not only reduces the stair-case effect but also replaces it with a smoother, glossier finish, thereby significantly enhancing the mold’s surface characteristics.

Incorporating CVS into the mold fabrication process offers significant advantages, particularly given the electroforming process’s sensitivity to mold surface quality. However, it is important to acknowledge that CVS may potentially affect the dimensional accuracy and intricacy of printed parts. Hence, selecting optimal conditions for CVS becomes imperative to mitigate these effects. Singh et al. [33] conducted an investigation into the impact of CVS on the dimensional accuracy of ABS printed parts and presented critical findings. To minimize these effects, they recommended the pre- and post-cooling of parts at 0 °C, maintaining the smoothing temperature at 50 °C, and utilizing cycles lasting less than 30 s. In line with these recommendations, the mold components underwent CVS for 15 s during two consecutive cycles, incorporating pre- and post-cooling to ensure minimal impact on the dimensions of the parts.

**Figure 8:**
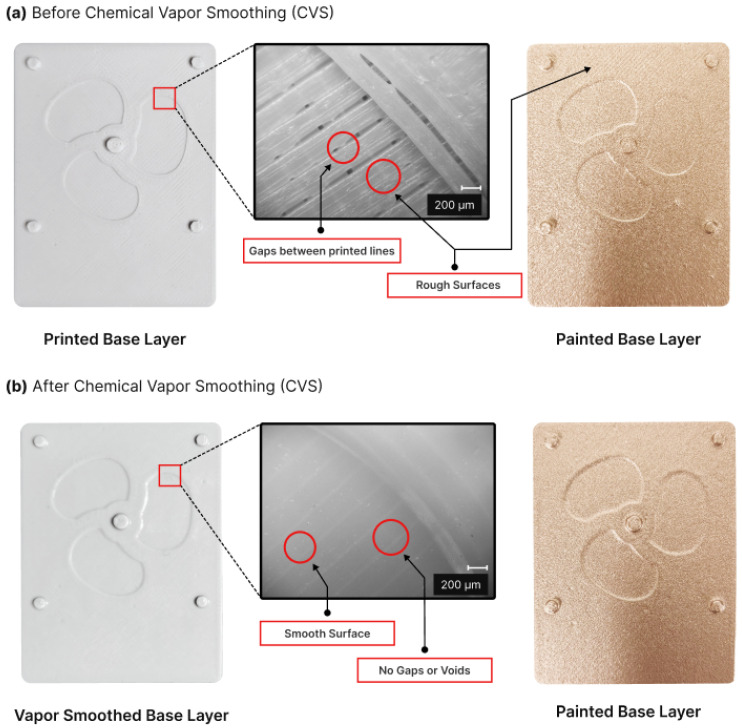
Images displaying the mold base layer of the toroidal propeller: (**a**) before chemical vapor smoothing, and (**b**) after chemical vapor smoothing.

Figure 8 offers a comparative view of the base layer of the toroidal propeller mold before and after vapor smoothing. In Figure 8a, the ABS printed base layer displays prominent printing lines, contributing to a rough surface characterized by peaks and valleys along each line. Microscopic examination exposed gaps between the printing layers, which could adversely affect the quality of the electroformed part within the mold. Despite the application of conductive paint, the printing lines remained visible in the painted base layer. Although the paint may have partially filled some gaps and reduced surface roughness, the overall texture remained relatively coarse, still indicating traces of the printing lines.

In contrast, in Figure 8b, the base layer is depicted after undergoing the CVS process. Remarkably, voids and gaps between the printing lines are no longer discernible. The CVS process effectively melted the printed layers, resulting in a considerably smoother surface. Microscopic inspection revealed that the vapor-smoothed base layer exhibited a significantly enhanced surface finish, free from any remnants of the original printing lines.

The impact of Chemical Vapor Smoothing (CVS) on mold dimensions was found to be remarkably minimal. Precision assembly of the mold, facilitated by locator pins, resulted in components fitting seamlessly after vapor smoothing, retaining their original configuration. However, a notable drawback of CVS is its tendency to induce warping in vapor-smoothed components, particularly those with thin profiles. To address this issue, the thickness of the base layer was increased from 1.5 mm to 3.5 mm, ensuring the stability of the mold design. Importantly, this adjustment in the base layer thickness did not affect the overall dimensions of the mold, as its primary function was to shape the toroidal propeller without impacting the object’s dimensions.

## 4. Results and Discussion

### 4.1. Multi-Level Object Electroforming Results

After the multi-layered mold of the center wheel was prepared, it underwent the electroforming process. The desired deposition thickness was 1600 μm, aligning with the center wheel design specifications. To reach this thickness, 32,000 deposition cycles were performed. The electroforming process lasted around 5 days, totaling 120 h, until the mold finished the designated cycles.

**Figure 9:**
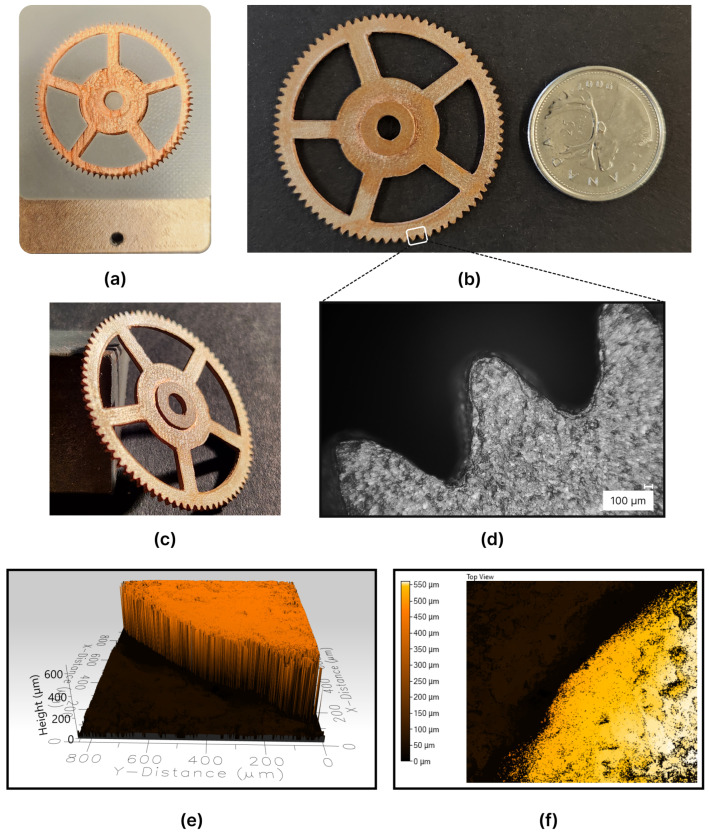
(**a**) The center wheel mold post-electroforming. (**b**) The resulting multi-level center wheel. (**c**) An isometric photograph displaying the center wheel. (**d**) Microscopic view of the center wheel’s teeth. (**e**,**f**) Optical scans revealing the center wheel’s hub.

After the electroforming process, the electroformed mold, depicted in Figure 9a, underwent a cleaning procedure using de-ionized water, followed by air drying. Inspection of the deposited material surface unveiled noticeable irregularities stemming from the non-uniform electroforming deposition process. These irregularities encompassed waviness, bumps, and microslits, underscoring the impact of process parameters on the magnitude and distribution of these surface characteristics arising from electroforming.

Furthermore, the electroforming setup is currently in its preliminary stage and would benefit from several enhancements. These upgrades entail integrating a filtration system, optimizing electrolyte circulation, and introducing a covering system to reduce the presence of debris and contaminants in the electrolyte. Despite these surface imperfections, the process effectively accomplished the fabrication of a structure with an approximate thickness of 1.6 mm. This achievement is noteworthy, considering the inherent difficulties associated with electroforming a structure of such thickness.

These results affirm the significance of surface post-treatments in attaining a controlled, uniform, and reproducible surface morphology in electroformed structures. Considering the sensitivity of most mechanical failures to surface properties, it is expected that improving the performance and functionality of electroformed components can be greatly enhanced by controlling surface topographical characteristics. Therefore, the adoption of surface post-processing technologies becomes crucial in enhancing the functionality and durability of electroformed structures. Consequently, mechanical surface treatments like grinding and polishing were incorporated into this manufacturing process to eradicate surface irregularities on the topmost surface.

The grinding process commenced with the use of 220 grit sandpaper to eliminate surface irregularities and unveil the desired intersection. Subsequently, the sample underwent consecutive polishing stages with 600, 800, and 1200 grit sandpapers to attain a smoother surface finish. Upon completion of the polishing process, both the mold and the electroformed part were submerged in acetone to aid in the separation of the center wheel from the mold. This separation procedure lasted about 2 h for the complete dissolution of the mold in the acetone.

The center wheel underwent an additional cleaning step using acetone to remove any residual paint particles. The bottom surface of the electroformed center wheel is illustrated in Figure 9b. As expected, the electroforming process reproduced the surface quality of the mold, yielding a bottom surface free from irregularities. However, some minor defects persisted in the part, mainly originating from the printing lines and dots inherent in the FDM-printed mold components, which are mirrored in the electroforming process.

The image of the center wheel captured at an angle, as depicted in Figure 9c, illustrates that the electroforming technique employed in AM-manufactured molds successfully generated the center wheel with varying thicknesses throughout its depth, thus realizing the intended multi-level structure. Initially, the extended hub of the center wheel was electroformed, followed by the gear body conforming to the mold’s contours to achieve the final center wheel shape.

Figure 9d provides a detailed examination of the center wheel teeth, observed through a digital microscope (VHX-1000, Keyence, Mississauga, ON, Canada). The teeth of the center wheel closely resembled the shape of the ABS mold. Upon microscopic inspection, differences in dimensions between the designed and fabricated geometry became apparent. Additionally, variations in fabrication quality were noticeable among different teeth. This expected variance could be attributed to the dimensional accuracy and inherent geometrical limitations of the FDM printer utilized in this study.

Furthermore, a segment of the center wheel hub underwent scanning using a 3D optical profilometer, Profilm 3D^®^, which employs white-light interferometry to measure surface profiles and roughness down to 0.05 µm. The optical scanner generated a visual representation of the bottom surface of the electroformed structure. The scanned area is depicted in Figure 9e. This scan revealed the copper growth within the two layers of the center wheel. Additionally, it indicated that the extended hub had a thickness of approximately 560 µm, deviating by around 40 µm from the intended thickness.

**Figure 10:**
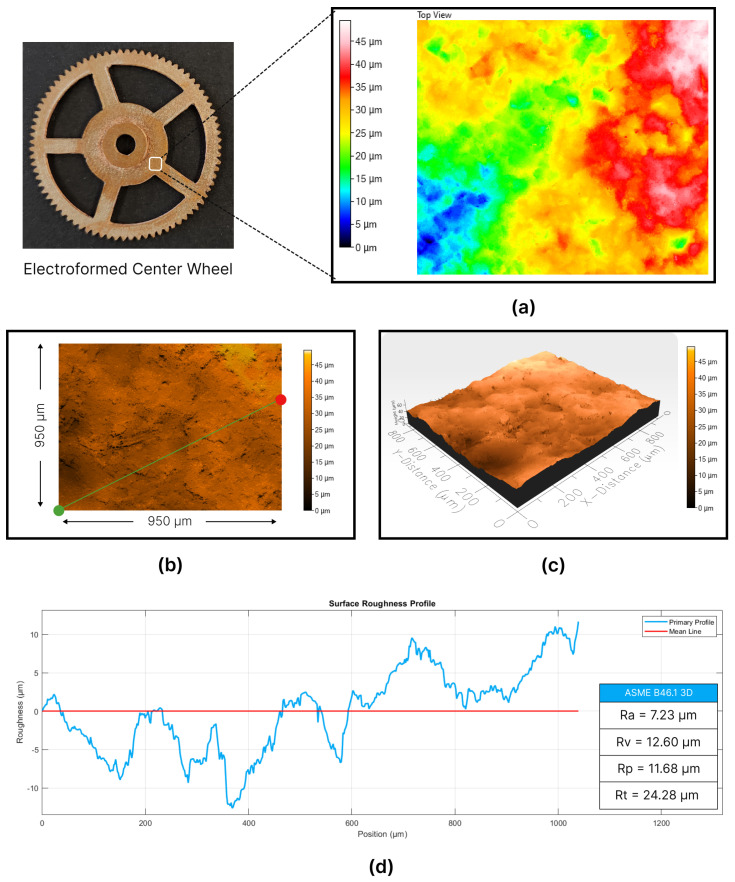
Surface topography analysis of the manufactured center wheel: (**a**) contour map, (**b**,**c**) representations of top and isometric surface profiles, and (**d**) roughness profiles.

To evaluate the manufacturing technique, surface roughness measurements were conducted on the bottom surface of the center wheel. The objective was to assess the quality of the as-deposited copper within the AM mold. Subsequently, surface topography and roughness measurements were performed using the Profilm 3D^®^ optical scanner. The specific area scanned, outlined in Figure 10, was a square approximately 950 µm in side length.

Contour maps serve as a valuable tool for visually representing the characteristics of the bottom surface (as-deposited surface) of the center wheel. As illustrated in Figure 10a, this contour map effectively displays elevation differences across the surface. Dark blue regions indicate lower elevations, while lighter red areas signify higher elevations. The elevation difference spans approximately 45 µm, indicating variations in surface height. Notably, a significant portion of the measured area falls within the range of blue, yellow, and green colors, indicating elevations between 15 µm and 40 µm. This range predominantly reflects the waviness of the surface, accounting for roughly 25 µm.

To further validate these findings, the top and isometric surface topography representations, depicted in Figure 10b,c, offer a detailed insight into the surface characteristics. These visual representations provide additional clarity regarding the overall quality and uniformity of the surface, reinforcing the observations obtained from the contour map analysis. Furthermore, the roughness profile, displayed in Figure 10d, offers a comprehensive overview of the surface variations along a defined path of 1050 µm. The path, indicated in the top surface topography figure, begins at the red dot and concludes at the green dot.

From the analysis of the roughness profile, several critical roughness parameters were derived in accordance with the ASME B46.1 3D standard. The mean arithmetic roughness, represented as $R_{a}$, was computed to be 7.23 µm. This parameter serves as an indicator of the average roughness across the surface. Two other significant parameters included the profile valley depth ($R_{v}$), measured as 12.60 µm, and the profile peak height ($R_{p}$), which was determined to be 11.68 µm. These values reflect the depths of the valleys and the heights of the peaks in the surface profile, respectively. Additionally, the maximum peak to valley height ($R_{t}$) was obtained by summing $R_{v}$ and $R_{p}$, resulting in a value of 24.28 µm. $R_{t}$ represents the maximum height difference between the peaks and valleys on the surface, offering a comprehensive assessment of the surface irregularities.

In conclusion, the electroforming process applied to the center wheel, notable for its multi-level configuration, achieved overall success. However, surface defects and irregularities observed on the top surface underscored the importance of mechanical surface treatment to achieve high-quality components. Conversely, the as-deposited bottom surface exhibited acceptable roughness levels, with minor waviness, rendering it comparable to surfaces produced through traditional machining methods. It is noteworthy that the FDM-printed mold components exhibited issues related to dimensional and geometrical accuracy, suggesting that employing higher-quality printing techniques could yield significant improvements. These findings underscore the potential for refining the electroforming process for manufacturing such intricate structures.

### 4.2. Free-Form Object Electroforming Results

Following the preparation of the free-form mold for electroforming the toroidal propeller, illustrated in Figure 7c, the mold was subjected to the electroforming process. The target deposition thickness was set to 1500 μm, in line with the toroidal propeller’s design specifications. To achieve this thickness, 30,000 deposition cycles were initiated.

The electroforming process extended over approximately 4.6 days, totaling 110 h, until the mold completed the designated cycles, with copper filling the mold completely. Post-electroforming, the electroformed mold underwent meticulous cleaning with de-ionized water followed by air drying. To minimize surface irregularities, the same polishing procedure was employed until the intersection between the mold and the deposited material was distinctly revealed.

**Figure 11:**
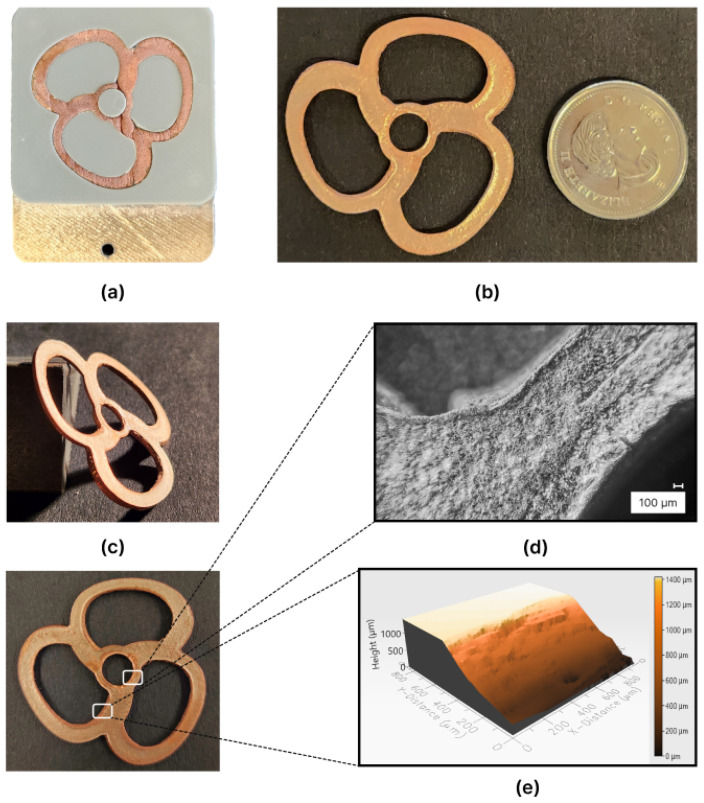
Images depicting the outcomes of electroforming the toroidal propeller: (**a**) the toroidal propeller mold post-electroforming, (**b**) a captured image of the produced toroidal propeller. (**c**) An isometric photograph showing the toroidal propeller. (**d**) Microscopic photo of the toroidal propeller, and (**e**) a profile scan of a toroidal propeller blade.

Figure 11a depicts the mold post-electroforming. Copper deposition adequately covered most of the mold surface, with certain areas exhibiting excessive thickness. However, voids were noticeable around the toroidal propeller hub. These voids and surface irregularities were mitigated through the post-electroforming polishing procedure applied to the mold. Subsequently, a similar immersion process in acetone to that used for the center wheel mold was performed, with the addition of heat and stirring using a magnetic stirrer. Remarkably, the part separation time was significantly reduced to just 45 min, compared to the approximately 2 h required for separating the center wheel from its mold.

Figure 11b,c showcases the electroformed toroidal propeller. The electroforming process adeptly replicated the intricate free-form structure with remarkable precision, particularly evident in the successful copper deposition within the intricate areas of the blades. In Figure 11d, a closer examination of the interface between the toroidal blade’s propeller hub and the blade is presented, captured using a digital microscope (VHX-1000, Keyence). This microscopic inspection underscored the electroforming process’s capability to intricately follow the curvatures of the toroidal propeller design, achieving a high degree of accuracy. Additionally, a segment of the toroidal blade underwent scanning using Profilm 3D^®^ to visually represent the curvatures and varying angles of the toroidal propeller blade. The optical image obtained is featured in Figure 11e.

**Figure 12:**
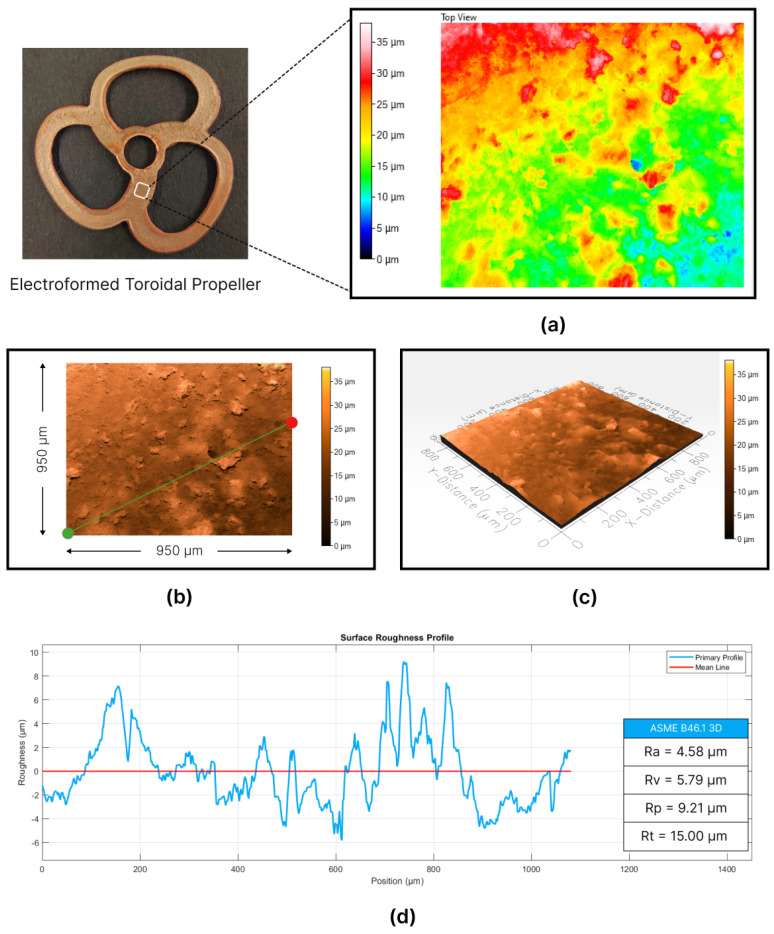
Surface topography analysis of the fabricated toroidal propeller: (**a**) contour map, (**b**) top surface profile representation, (**c**) isometric surface profile representation, and (**d**) roughness profiles.

Assessing the surface topography of the toroidal propeller was crucial for comparing the effects of CVS on the mold components, specifically the base layer responsible for forming the bottom surface. This evaluation utilized the Profilm 3D^®^ optical scanner for both surface topography assessment and roughness measurements. The scanned area on the bottom surface of the toroidal propeller, depicted in Figure 12, represented a square with a side length of 950 μm.

The surface contour maps and topographical representations, shown in Figure 12a–c, offer insights into the surface waviness and deviations within the scanned region of the toroidal propeller. The elevation difference spans approximately 35 µm, indicating variations in surface height. Moreover, a significant portion of the scanned region displays yellow and green colors, indicating elevations ranging from 15 μm to 30 μm. This color range predominantly represents surface waviness, accounting for approximately 15 μm, which is notably reduced compared to the center wheel.

The roughness profile, illustrated in Figure 12d, offers a comprehensive representation of surface variations along a defined 1050 μm path, marked in the top surface topography figure, starting at the red dot and ending at the green dot. The mean arithmetic roughness ($R_{a}$) was calculated to be 4.58 μm. Additionally, the profile valley depth ($R_{v}$) measured 5.80 μm, while the profile peak height ($R_{p}$) was determined to be 9.20 μm. Consequently, the maximum peak to valley height ($R_{t}$) was derived by summing $R_{v}$ and $R_{p}$, resulting in a value of 15.00 μm.

The surface roughness assessment results underscored a notable enhancement attributed to CVS, leading to a remarkable 37% improvement in surface roughness, decreasing from 7.23 μm to 4.58 μm. This level of roughness aligns competitively with the standards typically achieved through CNC machining [35,36]. Moreover, visual and microscopic examinations demonstrated that the toroidal propeller exhibited reduced influence from the printing lines and dots stemming from the FDM printing of the mold components. CVS effectively mitigated these imperfections, resulting in smoother and more uniform printed surfaces.

In conclusion, the findings outlined in this section underscore the electroforming technique’s versatility in crafting composite structures, combining both hollow and solid features, across multi-level and free-form configurations. The utilization of multi-layered mold components, coupled with the strategic application of conductive paint, evidenced the successful electroforming of diverse multi-level structures boasting multiple tiers in additive manufacturing molds. Moreover, the integration of modified angles and contours in mold designs facilitated the electroforming of intricate free-form structures. The employment of Chemical Vapor Smoothing (CVS) emerged as a valuable asset, effectively melting printing lines and yielding smoother surfaces for mold components, thereby amplifying the overall quality of the electroformed parts within the mold.

## 5. Conclusions and Future Outlook

The objective of this study was to develop design protocols for molds, primarily intended for electroforming multi-level and free-form metal components. These molds were manufactured using additive manufacturing techniques, aiming to facilitate the electroforming of customized miniature composite metal parts with various shapes and configurations. The research encompassed the development of a modular mold design and the formulation of experimental mold assembly procedures. The key discoveries and implications of this investigation are outlined below:

- Versatile mold design: The mold design was modular to cater to electroforming needs across diverse geometries, encompassing 2D, multi-level, and free-form solid as well as composite structures. Fabricated affordably through FDM printing with ABS filaments, these molds possessed sacrificial attributes, streamlining the part separation process following electroforming.
- Integrated methodology for mold preparation and electroforming: this systematic approach guided the entire workflow, encompassing mold manufacturing, conductive coating application, mold layers’ assembly, electroforming, and subsequent mechanical surface treatments utilized to the electroformed components.
- Implementation and enhancement of the proposed technique: The electroforming process successfully manufactured composite structures in both multi-level and free-form configurations, exemplified by the fabrication of a center wheel and a toroidal propeller. Achieving an average thickness of 1500 μm via electroforming posed a notable challenge, yet the resulting parts exhibited high quality, featuring minimal waviness on the bottom surface ($R_{a}$ = 7.23 μm) and a refined top surface post-mechanical treatment. The integration of Chemical Vapor Smoothing (CVS) to the mold components effectively eliminated printing lines and dots, consequently reducing roughness by 37%, thereby enhancing the overall part quality.

In conclusion, electroforming within additively manufactured molds presents a cost-effective way to produce metal parts with diverse geometric configurations. This approach not only offers higher-quality parts, particularly those with limited thickness, but also proves to be more economically viable compared to traditional metal additive manufacturing methods. Electroforming emerges as a financially advantageous alternative, encompassing both setup and production costs. Furthermore, its potential for parallelization enhances efficiency, especially in the context of personalized batch processing.

In the existing literature, electroforming has primarily been associated with thin-walled hollow structures, 2D components, and nanoscale applications. However, this study successfully demonstrated electroforming’s adaptability in producing solid and composite structures, spanning multi-level and free-form objects across micro- and macroscales. Significantly, the applications investigated in this research featured thicknesses exceeding 1000 µm, a notable challenge for electroforming due to difficulties in achieving uniform surfaces at such thicknesses. The methodologies employed in mold design and experimental applications not only address this challenge but also open avenues for mass personalization across diverse applications. This underscores electroforming’s potential to evolve into a reliable, robust, and versatile metal additive manufacturing technique.

As this study draws to a close, it paves the way for future exploration in the realm of electroforming as an additive manufacturing technique. The investigation of advanced FDM printing technologies that integrate ABS with conductive filaments is highly promising. In addition, multi-head FDM printers that are capable of extruding mold components in both ABS and conductive filament layers simultaneously offer heightened flexibility and precision in mold fabrication. To harness this potential, the selection of a highly conductive filament is crucial to facilitate electroforming. This approach not only improves mold quality but also facilitates the creation of intricate and complex metal parts.

Moreover, the integration of advanced additive manufacturing (AM) technologies, such as digital light processing (DLP), has the potential to significantly enhance precision and dimensional accuracy, surpassing FDM capabilities by a factor of five. This advancement opens avenues for producing precise and intricate structures, particularly microparts, thereby pushing the boundaries of mold fabrication and electroforming.

The efficiency of the electroforming process can be significantly improved by refining the current setup. Introducing a filtration system, optimizing electrolyte circulation, and reducing contaminants in the electrolyte with a covering system have the potential to enhance process predictability, efficiency, and the production of high-quality results.

Moreover, the scope of electroforming can be expanded by investigating alternative conductive materials like titanium, graphite, steel, and various alloys. These materials can fulfill the needs of applications that require durability, strength, and rigidity, thereby broadening the array of compatible materials for electroforming. Finally, exploring the mechanical characteristics of electroformed parts provides insights into their structural integrity and mechanical behavior. Such studies can significantly impact the design, application, and overall performance of electroformed components.

## Figures and Tables

**Table 1:** Parameters of FDM printing.

Parameter	Value	Unit
Print speed	30	mm/s
Layer thickness	0.1	mm
Initial layer Thickness	0.2	mm
Infill density	50	%
Wall thickness	1	mm
Extrusion temperature	245	°C
Build plate temperature	95	°C
Nozzle diameter	0.4	mm

## Data Availability

The original contributions presented in the study are included in the article, further inquiries can be directed to the corresponding author.

## Abbreviations

The following abbreviations are used in this manuscript:

ABS	Acrylonitrile Butadiene Styrene
AM	Additive manufacturing
CAD	Computer-aided design
CVS	Chemical Vapor Smoothing
FDM	Fused Deposition Modeling
LIGA	German acronym for lithography, electroforming, and molding
MP	Mass personalization
